# Clinical Application of Different Liquid Biopsy Components in Hepatocellular Carcinoma

**DOI:** 10.3390/jpm14040420

**Published:** 2024-04-15

**Authors:** Jing Xu, Yuanyuan Zhao, Zhishui Chen, Lai Wei

**Affiliations:** Institute of Organ Transplantation, Tongji Hospital, Tongji Medical College, Huazhong University of Science and Technology; Key Laboratory of Organ Transplantation, Ministry of Education; NHC Key Laboratory of Organ Transplantation; Key Laboratory of Organ Transplantation, Chinese Academy of Medical Sciences, Wuhan 430030, China; jingxx@hust.edu.cn (J.X.); yyzhao@tjh.tjmu.edu.cn (Y.Z.); zschen@tjh.tjmu.edu.cn (Z.C.)

**Keywords:** hepatocellular carcinoma, liquid biopsy, liver transplantation, circulating tumor cells, circulating tumor DNA, exosomes

## Abstract

Hepatocellular carcinoma (HCC) is the most common form of primary liver cancer, usually occurring in the background of chronic liver disease. HCC lethality rate is in the third highest place in the world. Patients with HCC have concealed early symptoms and possess a high-level of heterogeneity. Once diagnosed, most of the tumors are in advanced stages and have a poor prognosis. The sensitivity and specificity of existing detection modalities and protocols are suboptimal. HCC calls for more sophisticated and individualized therapeutic regimens. Liquid biopsy is non-invasive, repeatable, unaffected by location, and can be monitored dynamically. It has emerged as a useable aid in achieving precision malignant tumor treatment. Circulating tumor cells (CTCs), circulating nucleic acids, exosomes and tumor-educated platelets are the commonest components of a liquid biopsy. It possesses the theoretical ability to conquer the high heterogeneity and the difficulty of early detection for HCC patients. In this review, we summarize the common enrichment techniques and the clinical applications in HCC for different liquid biopsy components. Tumor recurrence after HCC-related liver transplantation is more insidious and difficult to treat. The clinical use of liquid biopsy in HCC-related liver transplantation is also summarized in this review.

## 1. Introduction

Primary liver cancer is the sixth most common cancer [1], and hepatocellular carcinoma (HCC) is the most common histological category, accounting for 70–85% of the morbidity in primary liver cancer patients [2]. Statistically, HCC is the second most fatal malignancy after pancreatic cancer, its five-year survival rate is approximately 18% [3]. The morbidity and mortality of HCC has continued to increase worldwide in recent years compared to other common cancers (e.g., breast, lung and prostate cancer). Frustratingly, the holistic prognosis of HCC continues to be very poor, owing to the concealment of symptoms in the early stage, the lack of validated early detection instruments, restricted therapeutic alternatives and the high recurrence rate [4].

**Figure 1:**
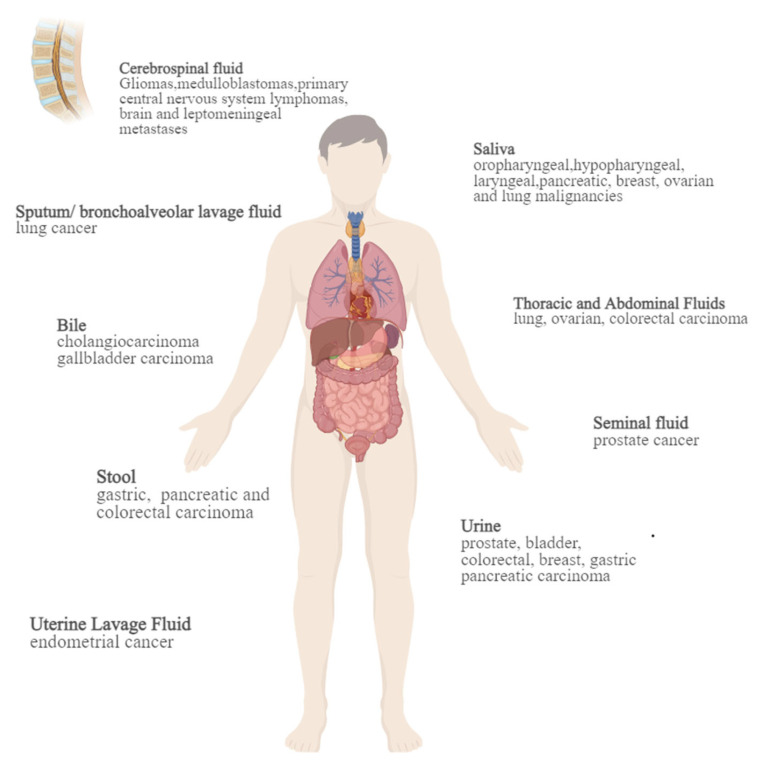
Liquid biopsies of non-blood origin in different malignancies.

With the perspective of the individuation and precision therapy of oncology taking root, liquid biopsy has gained the limelight. Blood is the most broadly used type of liquid biopsy specimen in medical research. In addition to blood, body fluids such as saliva [5], sputum [6], cerebrospinal fluid [7,8,9,10,11], bile [12], urine [13,14,15,16,17,18,19,20,21], stool [22,23,24], seminal fluid [25], and uterine lavage fluid [26] have also been explored in a variety of malignancies (shown in Figure 1). There are many substances that can be used as liquid biopsy targets, including circulating tumor DNA (ctDNA), circulating miRNA, exosomes, and tumor-educated platelets (TEP), in addition to circulating tumor cells (CTCs) and circulating cell-free DNA (cfDNA), which are more widely used. Liquid biopsy has been clinically approved for the diagnosis, treatment efficacy evaluation, and prognosis assessment of a variety of malignancies [27]. For example, CTCs are approved by the US Food and Drug Administration (FDA) as a prognostic assessment for breast, prostate, and colon carcinomas. Whereas, cfDNA is licensed for the adjuvant detection of non-small cell lung cancer (NSCLC), breast, and ovarian carcinomas.

This review aims to summarize the available enrichment techniques and clinical research on liquid biopsy from blood samples. With the growing number of HCC-associated liver transplant patients, tumor recurrence surveillance for this subset of patients is also an important factor affecting the patients’ overall survival. We specifically summarize clinical studies related to liquid biopsy in HCC-related liver transplant patients.

## 2. Application of Liquid Biopsy in HCC

**Figure 2:**
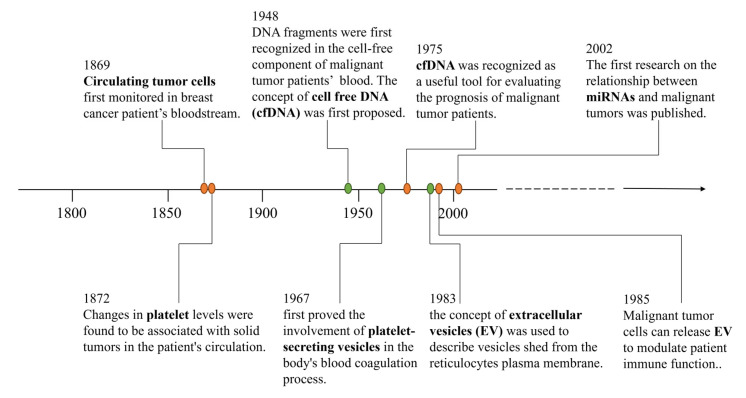
Historical time-line of the development of liquid biopsy components.

Since 1869, when the first detection of circulating tumor cells in breast cancer patients’ blood was made by Thomas Ashworth, the exploration of liquid biopsy has been ongoing for years (shown in Figure 2). Theoretically, liquid biopsy is non-invasive, repeatable, has good patient adherence, without the limitation of the biopsy site and allows for dynamic observation. Moreover, the spatial and temporal heterogeneity of tumor development can be surmounted by repeated follow-ups [28]. Thus, it is straightforward to understand the patient’s tumor progression and even therapeutic effects. Liquid biopsy holds great promise for screening, diagnosis, therapeutics and the monitoring of HCC patients.

### 2.1. CTCs

Carcinoma cells shedding from primary or metastatic tumors and circulating in body fluids are called CTCs. They are the earliest detectable markers that can be used for biopsy. CTCs are widespread among malignant tumor patients but extremely rare in healthy individuals or benign tumor patients [29]. In accordance with the previous findings, most CTCs are cleared upon entering the peripheral circulation, and only a small percentage of CTCs are capable of surviving in the peripheral circulatory system [30]. The surviving CTCs may come from various locations within the tumor and have different physiologies, and their capabilities of survival, invasion and metastasis vary [31]. In the seed soil hypothesis, the circulatory CTCs are considered the seeds of tumor metastasis [32].

The CTC enrichment approaches are numerous. Physical property-based assays [33,34,35,36] are convenient and fast, but CTCs have high variability in size. Their physical properties overlap significantly with leukocytes. So, this class of assays is less specific. The widest application currently is using immunoaffinity separation techniques for CTC enrichment. In Table 1, we exemplify the current CTC enrichment techniques and the respective advantages and disadvantages of each of them. Among these assays, the Cell Search System represents the only FDA-approved assay available in malignant tumor patients. Epithelial mesenchymal transition (EMT) is a phenotypic change characterized by epithelial property loss and mesenchymal property gain. Due to the role of EMT in tumor progression and metastasis [37], CTCs in malignant patients can be classified into three sub-groups: epithelial CTCs (E-CTCs), mesenchymal CTCs (M-CTCs) and intermediate subtypes undergoing transformation CTCs (E/M-CTCs) [38]. Hence, circulating CTCs in Malignant tumor patients who are undergoing transformation or have completed EMT do not represent the epithelium-derived specific marker, Epithelial cell adhesion molecule (Ep CAM) [39]. The CTCs in HCC patients have also been demonstrated to undergo EMT while in circulation [40]. This leads to a decline in Cell Search System monitoring sensitivity [41].

CTCs are currently widely used in therapeutic modality selection, treatment evaluation and recurrence monitoring of HCC. Jun Yan et al. demonstrated a significant relationship between tumor size and CTC number in an in situ mouse model of HCC, and the number of CTCs was also strongly connected with the treatment response [63]. Consistent results were also reported in HCC patients. CTC counts were significantly correlated with higher serum AFP levels, multiple lesions, more advanced TNM and BCLC stages, and embolisms or micro-embolisms [64]. The research by Lu-Nan Qi et al., which evaluated the role of CTCs in the diagnosis and prognosis of HCC patients, confirmed that CTC counts were substantially higher in advanced HCC patients with Barcelona Clinic Liver Cancer (BCLC)B–C stage than in early stage (BCLC0~A stage). Unexpectedly, two patients with HBV who were included in the same study as controls displayed low levels of CTC positivity and had a confirmed diagnosis of HCC at follow-up [65]. Malignant tumors metastasize in parallel with the development of the primary tumor [66]. CTC counts could serve as a promising indicator for HCC metastasis early diagnosis and disease progression assessment. This conclusion was evidenced in a retrospective study involving 195 patients with HCC [67]. Although there are no trustworthy prospective trials applying CTC for HCC screening, it is theoretically expected. CTCs may also have some guidance on how to maximize patient benefit by selecting from anatomical resection (AR) and non-anatomical resection (NAR). The retrospective study by Lu-Nan Qi et al. on 136 HCC patients treated with R0 resection pointed out that pre-operative CTC analysis could be used to improve the choice of resection procedure for HCC patients, and that AR only improved patient survival in those with low CTC counts and negative M–& E/M-CTC phenotypes [68]. In addition to the surgical choice of liver resection, CTC can also provide pre-operative guidance on the best surgical margins before liver resection. Pre-operative CTC status can also predict the magnitude of microvascular infiltration (MVI) in HCC patients before obtaining laboratory pathology results, thus guiding therapeutic and evaluating prognosis [69]. The amount of CTCs changes over time with the treatment process; therefore, theoretically, the dynamic observation of CTC levels can assess the treatment effect and monitor recurrence. CTC load decreases shortly after resection, and a higher predisposition to tumor recurrence is demonstrated by patients with persistently high post-operative CTC load [70]. Yun-Fan Sun et al. discovered that post-operative CTC count was a predictor of extrahepatic metastasis after curative surgical resection in a retrospective training cohort of 144 HCC patients and this was confirmed in a prospective validation cohort of 53 patients [71].

CTCs possess an intact cellular structure, can fulfill cellular morphology and function research under in vitro conditions, and can even be cultured or developed in vitro. By using CTCs, it is possible to forecast the reaction or resistance of tumor cells to given treatments in vitro and direct individualized programs. The nucleic acids and proteins contained within them can also be analyzed for corresponding histology [72]. Nevertheless, the CTC expression is extremely low in the blood [73] and existing enrichment strategies are not able to fulfill the clinical requirements. No site-specific markers have been found to be stably expressed in HCC patients CTCs [74]. Two systems, the Cell Search System and Can Patrol System [65] still need trustworthy prospective studies to compare their effects on HCC patients. Additionally, there are a few studies related to the use of CTC testing in patients undergoing nonradical therapies, and the future of the use of this liquid biopsy type in such patients is unknown. Existing studies in HCC patient cohorts are scattered and lack consistency, making it impossible to compare the outcomes. Despite the theoretical feasibility of overcoming tumor size and heterogeneity, CTC detection remains only an indirect method in the management process of HCC patients. Large clinical trials are needed to demonstrate this in the future.

### 2.2. cfDNA & ctDNA

Mandel and Metais identified fragmented DNA in the cell-free component of blood circulation in cancer patients in 1948, giving rise to the concept of cell-free DNA (cfDNA). Thereafter, cfDNA did not receive adequate focus until 1975. Leon et al. concluded that cfDNA could be applied to assess the prognosis of malignancy patients. CfDNA is a typically double-stranded fragment of approximately 150–200 base pairs in length. Free DNA fragments in circulation have a relatively complex origin. In healthy individuals, circulating cfDNA is predominantly originated from hematopoietic cells, especially leukocytes, and it is typically at low steady-state levels [75,76,77]. In the circulation of malignancy patients, the fluctuating intensity of cfDNA ranges widely [78]. The abnormalities of cfDNA in malignant tumor patients include both quantitative and qualitative changes. Quantitative changes refer to elevated levels of cfDNA in malignancy patients compared to healthy individuals or benign disease patients. When patients with malignant tumors receive treatment, the serum levels will be further increased [79]. Qualitative changes include altered DNA integrity and tumor-associated genetic variants such as specific point mutations and DNA methylation. Circulating tumor DNA (ctDNA) is a portion of cfDNA that expresses specific somatic mutations associated with the development of malignant tumors, accounting for approximately 1% of the total [80]. The overwhelming proportion of ctDNA is not generated from CTCs [81].

There are two assays currently in use. One is using PCR techniques to detect some mutations already known, including real-time fluorescence quantitative PCR (qPCR), droplet digital PCR (dd PCR), etc. The other is using non-targeted techniques to classify the detected DNA fragments (e.g., Whole Genome Sequencing (WGS), Sanger sequencing and Next-generation sequencing (NGS)). The former can not only magnify DNA fragments but also dynamically filter aberrations in DNA fragments with high sensitivity and low cost; however, only mutations in a pre-established panel of genes can be detected. The latter one can demonstrate the entire genomic map more thoroughly, but is relatively time-consuming and costly [82].

Ken Chen et al. compared serum cfDNA levels between HCC patients and healthy persons and found that serum cfDNA levels were positively linked to the HCC differentiation degree and metastasis but negatively correlated with patient prognosis. Also, serum cfDNA levels were relevant to survival after hepatectomy. To some extent, it has been indicated that cfDNA could be beneficial for HCC monitoring and could be used as a potential marker for decision-making on surgical or non-surgical treatment [83]. Korean research on post-radiotherapy monitoring of HCC patients also stated that the cfDNA levels of patients with pre- and post-therapy were significantly different, and the cfDNA levels measured immediately after radiotherapy could earlier forecast the partial therapeutic response [84]. Also, serum cfDNA levels have been demonstrated to be a prognostic indicator for extrahepatic recurrence after curative surgery in HCC patients [85]. In addition to variations in content, HCC patients showed abnormalities in the integrity of circulating cfDNA compared to healthy individuals or benign disease patients. A study by Ao Huang et al., using qPCR to detect cfDNA integrity in 69 HCC patients who underwent hepatectomy, stated that patients with HCC had a lower integrity of circulating cfDNA compared to healthy individuals or benign liver disorders, and the cfDNA integrity sharply increased after hepatectomy [86]. Research on circulating cfDNA integrity in HCC patients using parallel sequencing methods, nevertheless, denoted that free DNA fragments in HCC patients’ plasma existed at different lengths, both shorter and longer [87]. There is no controversy that the size of plasma DNA fragments varies depending on their origins. Atsushi Ono et al. investigated that the presence of ctDNA could indicate neoplastic progression and that ctDNA could be tested to anticipate portal vein invasion and neoplasm recurrence, especially extrahepatic metastases within 2 years. In addition, the results of this study also suggested that ctDNA showed a better and more sensitive outcome than traditional tumor markers in some patients [88]. Research on ctDNA, after all, has mainly focused on the analysis of its molecular characteristics. By analyzing whole-exome sequencing data from 11 HCC patients’ tumor samples, Geng Chen et al. proved that different phenotypes of clonal evolution do exist in the HCC patient population, and these phenotypes are significantly connected with patients’ clinical disease duration. Simultaneously, dynamic monitoring of ctDNA levels can mirror HCC load in real time and can be used to pursue tumor clonal evolution in HCC [89]. CtDNA has a short-term middle life and its content depends on tumor load, resulting in broader applications in detecting HCC recurrence and predicting prognosis. Prior to hepatectomy, the status of HCC patients’ ctDNA can be a predictor of tumor recurrence possibility, and the genetic analysis of ctDNA at multiple time points combined with tumor tissue genome sequencing can provide pinpoint information for tumor recurrence [90]. The mutations detected in circulating free DNA fragments can, to some extent, reveal the pool of tumor-associated gene mutations in patients, and therefore, the analysis of ctDNA can deliver a high degree of specificity [91,92,93,94]. In parallel, its quantity correlates with tumor load, the ctDNA is abundant in advanced HCC patients [95]. Johann von Felden et al. utilized mutational signatures of ctDNA to characterize the mutational landscape of advanced HCC patients in detail, filling the vacancy of lacking adequate specimens for biomarker investigations in advanced HCC patients and, to a certain extent, demonstrating again the clinical usefulness and application prospects of ctDNA molecular analysis [96]. But in fact, the amount of ctDNA is very minimal, so there are still missing credible clinical studies in the early diagnosis of HCC. However, some investigators have established a diagnostic prediction model including ctDNA by comparing tumor tissue and normal hematopoietic cell-derived cfDNA gene profiles [97]. This model achieves high diagnostic specificity and sensitivity for HCC tumor load, treatment response and staging, which, to a certain extent, reflects the potential of ctDNA in early diagnosis of HCC. Though free DNA fragment detection is represented in some studies as more sensitive than traditional tumor markers for HCC, more investigations suggest combining the two assays for better sensitivity and accuracy [83,98].

CfDNA/CtDNA correlation analysis yields a comprehensive tumor-associated mutation spectrum. It is more convenient for evaluating tumor heterogeneity, overseeing tumor kinetics, tracing genomic evolution, and monitoring the emergence of acquired drug resistance [95]. However, the tumor histological information carried by DNA fragments is incomplete [99]. This approach fails to recognize the abnormal expression of non-DNA-based alterations in patients. Certain biological elements may also affect the results of the measurements. It is still a perplexing puzzle as to how cfDNA/ctDNA is released into the bloodstream. The contribution of different cellular origins to their content during different physio-pathological processes, such as tumor progression, therapy response, and drug resistance is also not clear [100]. DNA fragments have a short circulating half-life [101], and the appropriate monitoring time needs to be further determined. Existing studies of cfDNA/tDNA analysis are not standardized, and the steps involved prior to analysis may affect the results of the measurements [94], thus the implementation of cfDNA/ctDNA in the clinical milieu remains limited.

### 2.3. Circulating microRNA

MicroRNAs are a category of homologous, evolutionary conserved, non-coding small molecules with a length of about 20~24 nucleotides. They mainly interact directly with the corresponding target messenger RNA (mRNA) at the post-transcriptional level to exert gene regulation [102,103]. The first report on the connection between miRNAs and malignancies can be dated back to 2002 [104]. As investigations intensified, miRNA signatures displayed remarkable differences according to tumor categories, thus establishing a great potential of miRNAs in the diagnosis, prognosis and therapeutic evaluation of malignant neoplasm patients [105]. For most malignancy patients, miRNAs have a two-fold function: being carcinogenic and tumor-suppressive. The expression of miRNAs in HCC patients’ neoplastic tissues also varied remarkably depending on their roles. In Table 2, abnormal miRNA expression changes in HCC tissues and their roles in the development of HCC are listed. MiRNAs in tissue specimens, despite demonstrating great promise for application are invasive. Circulating miRNAs appear to be a much safer and more effective option. There are two major origins of the extracellular milieu miRNAs, selectively exported by active cells or excreted as a byproduct of cellular activity and cell death [106]. Using integrated serum samples from prostate cancer patients and xenograft models, Patrick S. Mitchell et al. demonstrated that circulating miRNAs are present in the circulation with a high degree of stability. In addition to this, their study indicated that miRNAs have anti-plasma RNase activity in the circulatory system, the levels of miRNAs released into the circulatory system by malignant tumors are adequate for detection. Moreover, it is feasible to apply miRNAs as biomarkers regardless of both serum and plasma sources [107]. Based on this conclusion, circulating miRNAs are well-equipped to perform as circulating biomarkers for the detection of common human cancer species.

The methodologies employed to test circulating miRNAs include PCR, in situ hybridization, microarrays and RNA sequencing, etc. [130]. Quantitative PCR is still the most widely used one.

Serum miRNA-107 levels were significantly elevated in HCC patients compared to healthy volunteers and may be a viable marker for early HCC screening and diagnosis [131]. Circulating miRNA levels were analyzed in a group of hepatitis B virus (HBV) patients with pathologically confirmed dysplastic nodule (DN) and early HCC. miR-122 and let—7b levels exhibited significant differences in this cohort, indicating that miRNA can be used to identify early HCC from DN in patients with chronic hepatitis B [132]. Likewise, plasma miR-148a has demonstrated its ability to recognize HCC patients, cirrhotic patients and healthy individuals. It showed similar susceptibility and specificity to AFP in the diagnosis of HCC. The same applies to AFP-negative HCC patients. Circulating miR-148a levels progressively decreased during the process of cirrhosis to HCC and recovered after hepatectomy, expressing the clinically required sensitivity and specificity of the biomarker for neoplasia [133]. Previous studies have noted that different causes of HCC manifest different miRNA abnormalities [134]. This was reconfirmed in a study that correlated the premature prediction of hepatitis C virus with (HCV)-associated HCC patients. Nine miRNAs (miR-142, miR-150, miR-183, miR-199a, miR-215, miR-217, miR-224, miR-424 and miR-3607) were discovered to be differentially expressed in HCC and HCV patients compared to healthy volunteer serum specimens. The investigators combined miR-150, miR-199a, miR-224, miR-424 and miR-3607 together as an early detection instrument and it did show better sensitivity and specificity [135]. miR-497 and miR-1246 levels not only related to TNM stages and metastasis in HCC but also to some extent, could act as a prognostic predictor [118]. Besides its great promising application in the early diagnosis of HCC, miRNA has also been tried to appraise therapeutic efficacy and prognosis. In two cohorts comprised of HCC patients who underwent radiofrequency ablation (RFA), the Let—7c expression level proved to be a predictor of post-treatment tumor recurrence. Unfortunately, no clear predictive value for recurrence was found in the small group of HCC patients who underwent surgery in the same study [136]. Changes in circulating miRNA levels are equally relevant for surveillance in HCC patients receiving thermal ablation (TA) and transcatheter arterial chemoembolization (TACE) [137]. In the systemic therapeutic treatment of HCC, FDA-approved drugs are targeted at a single point. miRNAs can engage in key pathways of HCC development and progression by binding to multiple mRNAs, which means that miRNAs have the potential to be valuable therapeutically [138]. Douglas D. Young et al. first reported microRNA-related drugs in 2010. miR-122 inhibitors can reduce HCV virus replication in hepatocytes, while their activators can selectively cause apoptosis in hepatocellular carcinoma cells [139]. Since then, many miRNA regulators have been shown to have anti-tumor efficacy [140,141,142].

Circulating microRNA can comprehensively reflect changes in the post-transcriptional level of tumor cells. Its potential therapeutic value is more worthy of attention and in-depth study. There are numerous categories of miRNAs, and the same miRNA may correspond to several different targets [104]. The current studies are mostly retrospective in the laboratory, focusing on specific cases rather than broadly. Meanwhile, miRNA effects in the molecular pathogenesis of HCC at different stages have not been sufficiently investigated in depth. Further investigations are necessary to pinpoint the exact time when circulating miRNAs are detectable in the circulation during the genesis and progression of HCC.

### 2.4. Exosomes

The concept of the extracellular vesicle (EV) was first used to describe vesicles shed from the plasma membrane of reticulocytes in 1983 [143]. In 1985, Poutsiaka et al. demonstrated that malignant cells can down-regulate tumor–host immune function by releasing EVs [144]. It was later utilized to describe granules that are naturally released by cells into the extracellular space, these granules have a lipid bilayer structure similar to that of human cells but do not contain a nucleus hence they do not have the ability to self-replicate [145]. EVs are regarded as important and acids, proteins, lipids, and many other components are isolated within the bilayer membrane structure of extracellular vesicles, as well as organelles; all of those components play vital functions in multiple physiologic and/or pathologic pathways. It has many designations in published studies, including micro-vesicles, exosomes, nanoparticles, particles, etc. The classification was previously dominated by the biological origin of extracellular vesicles: micro-vesicles (100–1000 nm in diameter, derived from the plasma membrane), exosomes (30–100 nm in diameter, endosomal origin) and apoptotic vesicles (50–200 nm in diameter, released by apoptotic cells) [146]. The guidelines suggest that extracellular vesicles should be designated according to size, density, biochemical composition and cellularity in a detailed classification, on which there is no consensus [145]. For the sake of consistency, this review uses exosomes for description. Under the umbrella of the phospho-bilayer, the materials contained within the exosomes are in a separate environment, and therefore, have better stability than proteins, nucleic acids, and other substances that are individually present in the circulation [147]. Exosomes play a facilitating role in the development of liver disease, cirrhosis and HCC from various causes. Many proteins and nucleic acids contained within exosomes can serve as potential markers of HCC [148]. In Table 3, the components of exosomes associated with the process of HCC development, metastasis, and treatment are presented.

Isolation and enrichment techniques for exosomes include ultracentrifugation, ultrafiltration, immunoaffinity, polymer-based precipitation, size exclusion chromatography, ion exchange chromatography, microfluidic-based technologies and membrane-based isolation techniques [171]. Ultracentrifugation is still considered the gold standard for isolating exosomes till now [172].

By comparing the exosome mRNA in HCC patients with cirrhosis, chronic hepatitis B, and healthy individuals, the investigators found that mRNA detection within the serum exosomes has early HCC diagnostic value [173]. Similarly, the detection of exosomes lncRNA can be applied to HCV-related HCC early diagnosis and screening [174]. Other components share the same role, such as exosome miRNA-10b-5p [175]. Compared to circulating miRNAs, exosome miRNAs performed better. A prospective study in an HCC patient cohort undergoing curative surgery denoted that exosome miRNA-125b had a predictive function in post-operative HCC recurrence and long-term survival. Its low expression levels were associated with tumor numbers, cellular differentiation and TNM stage; the participants with lower levels exhibited shorter recurrence times and total survival [176]. Tomoyuki Suehiro et al. first reported that decreased levels of exosome miRNA-122 expression were associated with poor prognosis after TACE, and they also remarked that serum exosome miRNA-122 levels could reflect liver injury and residual liver function [177]. A similar finding was observed in the study of serum exosome circular RNAs (circ RNAs) in HCC patients. Circ CCAR1 expression was significantly correlated with vascular invasion, tumor size as well as TNM stage and differentiation grade, and HCC cells with high expression levels were resistant to anti-PD1 therapy [178]. Indeed, circulating exosome non-coding RNAs (ncRNAs) have a certain prognostic value and clinical application in HCC patients regardless of the treatment protocols adopted [179].

Compared to other new liquid biopsies, the stability of the exosome’s bilayer membrane structure and the unique capacity to be a “messenger” enable exosomes to participate in the HCC therapeutic schedule. Most chemotherapeutic drugs are toxic to both cancer cells and healthy cells, and their low specificity often causes undesirable toxicity and side effects. To avoid the normal cell or organ toxicity caused by the free diffusion of drugs, specific drug delivery systems have attracted the eyes of researchers. Compared with artificial synthetic nanodrug delivery materials, exosomes possess the merits of small size, easy accessibility, non-toxicity, organ- or cell-specific tropism, higher stability and bioavailability [180,181,182]. Furthermore, human cell-derived exosomes have many unique advantages that are hard to replace with synthetic materials. Their biocompatibility and low immunogenicity, coupled with the presence of bioactive membrane proteins on their surface, permit the specific delivery of goods to target cells without activating or attenuating the immunogenic response of the host’s immune system [183]. Even more importantly, exosome delivery enables their goods to penetrate the biological/physical barriers of the body (e.g., blood-brain barrier) [184,185]. Exosomes can participate in anti-tumor therapy in two manners: first, by using their capacity of material transportation, injecting exosomes that contain anti-tumor drugs or have oncogenic function in patients; second, by using the immunological properties of exosomes and triggering specific immune responses in tumor patients to achieve anti-tumor effects. Guohua Lou et al. in a nude mouse transplantation tumor model demonstrated that miRNA-122-transfected bone marrow mesenchymal stem cell exosomes could alter the expression of miRNA-122-target genes in HCC cells, thus sensitizing cancer cells to sorafenib by the communication with HCC cells and remarkably increasing the in vivo anti-neoplastic efficacy of sorafenib in HCC [186]. In a similar vein, bone marrow mesenchymal stem cell exosomes can serve as an efficient delivery vehicle for miRNA-199a. By targeting the mTOR pathway, it increases the susceptibility of hepatocellular carcinoma cells to chemotherapeutic agents effectively in an orthotopic HCC mouse model [187]. Apart from the relevant genes being loaded to improve the chemotherapy sensitivity of hepatocellular carcinoma, the chemotherapeutic drugs themselves can also be used as exosome “cargo”. Doxorubicin-loaded exosomes were developed. HCC cells and tumor stem cells (CSCs) showed significant cell uptake and cytotoxicity to the Doxorubicin-loaded exosomes. This conclusion has been confirmed in both subcutaneous transplantation tumor models, orthotopic tumor models and advanced metastatic tumor models [188]. This suggests that exosomes have the potential to act as drug carriers to enhance anticancer efficacy. Even more surprisingly, we can use exosomes to deliver multiple drugs simultaneously and achieve synergistic antitumor effects. Dongdong Wang et al. successfully combined photothermal therapy with low-dose chemotherapy by delivering radioactive particles (Bi2Se3) and chemotherapeutic drugs (doxorubicin) via exosomes. The therapeutic regimen produced significant synergistic anti-tumor effects. This study showed that synergistic treatment through an exosome drug delivery system can reduce the dose of anticancer drugs, thus effectively reducing the side effects and improving economic efficiency [189]. Using mesenchymal stem cell-derived exosomes to block the relevant pathways [190], as well as altering the expression levels of tumor-associated macrophage-derived exosomes miRNA-125a and miRNA-125b [191] can inhibit the malignant behavior of HCC cells in cellular studies. In an in situ HCC mouse model, the vaccine formed by modified exosomes generated dramatic tumor inhibition and even complete tumor eradication by inducing oncology-specific immune responses [192]. It does provide a scalable approach for personalized immunotherapy of HCC.

The exosomes are enriched in contents and stabilized in nature. Existing mature enrichment protocols are still inadequate to completely separate extracellular vesicles from circulation. Exosome isolation and purification technologies are still in the process of development, and there is currently no harmonized methodology or platform for clinical trials to be conducted [193]. Further exploration of whether there are interactions between exosome-rich contents is also needed. It has a very huge development potential as a targeted drug delivery carrier [194]. However, the drug delivery system involved in exosomes is still far from being ready for clinical use. In addition to isolation and purification, how to integrate the desired miRNAs into exosomes, and how to ensure that the exosomes are targeted to the correct organ or tissue. These issues need to be addressed urgently [195].

### 2.5. Platelets & TEPs

Platelets originate from mature megakaryocytes. They are alive for about seven to ten days in peripheral blood and present in abundant quantities [196]. There is no nucleus inside the platelets, but mitochondrial DNA exists in the cytoplasm; various RNA molecules and proteins can be inherited from megakaryocytes, either generated by themselves or obtained externally [197]. Beyond their essential role in physiological processes such as hemostasis and thrombosis, platelets have been found to be important participants in all steps of oncogenesis and neoplastic progression [198]. Increased platelet content associated with solid neoplasms was originally discovered by Leopold Riess in 1872 [199]. This phenomenon was subsequently noted in patients with a variety of cancers to correlate with poor prognosis [200,201,202,203]. A prospective cohort study of 40,000 patients in the United Kingdom showed that about 5% of cancer victims had increased platelet counts prior to diagnosis [204].

The majority of hepatocellular carcinomas develop from cirrhosis; splenomegaly and hypersplenism caused by hepatic fibrosis are often manifested in HCC patients as well; therefore, platelet count increase is hardly noticeable in HCC patients. The degree of thrombocytopenia is tightly correlated with liver functions. Raised platelet counts are connected with HCC progression. Platelet counts may have opposite predictive roles on HCC patients with different hepatic basal circumstances. In HCC patients with combined hepatocirrhosis, thrombocytopenia may indicate a poor prognosis. In contrast, in patients without cirrhosis, thrombocytosis is more frequently associated with a worrisome outcome [205]. A retrospective cohort study found a well-signed positive correlation between pre-therapeutic platelet count and extrahepatic metastasis of HCC [206]. Simultaneously, the study argued that the comparatively lower incidence of extrahepatic metastases in HCC patients could be partly facilitated by thrombocytopenia. Some researchers are skeptical about this conclusion [207]. A study performed in two large HCC cohorts, on the other hand, found that thrombocytosis was independently associated with high tumor load and poor prognosis [208]. Higher pre-treatment platelet levels may be associated with the more aggressive tumor type. On a similar note, contrary findings have been reported. Research by Sangbin Han et al. concluded that there was no correlation between platelet count and tumor aggressiveness. However, they also expressed that the risk of postoperative mortality after HCC-associated living donor liver transplantation increased with an elevated platelet count [209]. Pretreatment measurement of platelet counts in TACE-treated HCC patients may predict, to some extent, both the tumor response to treatment and the long-term prospects of the patient [210]. The relationship between platelet count and HCC patient prognosis is controversial [211].

When malignant tumors develop in the organism, platelets are also “educated” by tumor cells. During this dual interaction, the RNA and protein expression profiles in the platelet cytoplasm are altered accordingly. Therefore, they are also called “tumor-educated platelets” (TEPs) [212]. TEPs possess the potential for early cancer diagnosis [213], disease progression surveillance [214] and treatment response supervision [215]. TEPs are more specific than simple platelet count changes. The previously addressed novel biomarkers are low in early cancer stages. TEPs are abundant in circulation and with standardized isolation procedures, may be a superior candidate [197]. TGF-β, NF-κB, VEGF, AKT, and PI3K were selected as alternative biomarkers based on previous investigations, and blood platelets were tested in 20 confirmed HCC patients and 10 control samples [216]. All selected RNA biomarkers were sufficient to successfully separate HCC patients from controls. Amongst them, AKT and PI3K have good detection potential for early HCC. Likewise, mRNAs such as CTNNB1, Rho A, SPINK1, IFITM3, and SERPIND1 contained in TEPs could permit earlier diagnosis of HCC from cirrhotic nodules. This result was evidenced in a 50-person cohort comprised of healthy subjects, patients with HCC, and cirrhosis [217]. By the bioinformatic analysis of platelet miRNAs, 250 miRNAs with differential expression were identified in blood samples from HCC patients and controls. These miRNAs, such as miR-495-3p and miR-1293, may serve a role in the growth, development, and metastasis of HCC [218].

Simple platelet count changes are convenient to measure, but their significance in HCC patients is unclear. TEPs are characterized by high stability and richness in blood. Due to their “education” by tumor cells, RNA expression and proteomic analysis of TEPs are of special significance. TEPs, although highly feasible in theoretical terms, have too few studies available. Most of the studies focus on the application of HCC screening. In fact, the splicing mechanism in platelets may be distinct from that in cells [219]. The same miRNAs may have entirely divergent effects in platelets and cells. Thus, relevant research needs to be further refined.

## 3. HCC-Associated Liver Transplantation and Liquid Biopsy

Favored by the simultaneous resection of the primary tumor and malignant-prone cirrhotic milieu, liver transplantation (LT) has been the preferred therapeutic option for early-stage HCC. With the progress of HCC therapeutic protocols and satisfactory preoperative conversion therapy, the criteria for HCC patients’ transplantation selection are being worked on to expand them [220,221]. HCC-associated LT is increasing year by year, and the number of HCC recurrences after liver transplantation is increasing as well. Incidentally, in the post-transplant condition, the recipient’s normative and acquired immunity are suppressed, making HCC recurrence more insidious, aggressive, and difficult to control [222]. There are no consensus or best practice guidelines for cancer surveillance and recurrence management after HCC transplantation. Non-invasive and reproducible liquid biopsies may have enormous potential for post-transplant recurrence monitoring.

As mentioned earlier, more sensitive assays are needed for HCC-related liver graft recipients, due to earlier tumor staging, more complete treatment and insidious recurrence. Feng Xue et al. created an integrated subtractive enrichment and immunostaining-fluorescence in situ hybridization (I FISH) platform to detect circulating CTCs. Superior sensitivity was demonstrated in a cohort of HCC-related liver transplant patients and healthy volunteers. It also indicated that the level of CTCs in the peripheral circulation is a potential prognostic marker for HCC-related liver transplant patients [223]. In a prospective study conducted on 47 HCC-associated LT patients, CTC was tested in subjects’ blood samples from pre-operative, a month post-operative, and three months post-operative [224]. They argued that pre- and post-operative CTC assessment for LT was not statistically significant as a predictor of recurrence. However, CTC subtypes did change throughout the follow-up process. In the same vein, Yun-Liang Xie et al. argued that changes in the total number of CTCs pre- and post-liver transplantation could not be used to evaluate the postoperative prognosis in HCC patients. Yet, the positive M-CTCs or the elevated proportion of M-CTCs after transplantation were associated with postoperative recurrence. M-CTC positivity could be an independent risk factor. This subset of patients has a higher recurrence rate and shorter survival after LT [225]. Through miRNA analysis of pre-operative liver transplantation serum samples from four relapsed and two non-relapsed patients, exosome miRNA-718 and miRNA-1246 were found to be differentially expressed. Subsequently, in 59 preoperative serum samples from patients with HCC-related liver transplantation, the investigators discovered that exosome miRNA-718 could be employed as a biomarker for tumor recurrence monitoring [226]. Toshiaki Nakano et al. proved that circulating exosomes have a well-defined significance in HCC occurrence and development within an animal model. This is ground-breaking in the field of HCC. In parallel, they analyzed blood samples from living donor liver transplantation (LDLT) patients and demonstrated that the HCC patients with a higher risk of early post-transplant recurrence (<2 years) were accompanied by a high level of exosome miR-92b before LDLT. After transplantation, patient exosome miR-92b has favorable forecasting accuracy for early HCC recurrence. This biomarker does not have adequate power to predict late HCC recurrence [169].

The use of liquid biopsy in liver transplant patients goes beyond tumor recurrence monitoring. It aids in assessing graft function [227], ameliorating ischemia-reperfusion injury [228,229,230], and monitoring acute rejection [231,232,233].

## 4. Discussion

In comparison with lung, breast, and prostate cancers, HCC is highly heterogeneous. As a result, HCC is not only difficult to diagnose at an early stage, but its treatment modalities are more varied and its outcome is more unpredictable. This has led researchers to expect more on liquid biopsy. After all, liquid biopsy is full of theoretical feasibility.

Table 4 summarizes the use of different liquid biopsy components in HCC diagnosis and treatment. While it is far more well-studied in other cancers, detection accuracy also limits the clinical use of liquid biopsies. Only a few components have been approved by the FDA for clinical usage in other cancers. In this regard, the development of liquid biopsy relies on more sensitive and specific enrichment protocols. The clinical significance of this technique in HCC patients is still in an advanced stage of exploration. The majority of existing studies on HCC are cellular or animal studies in the laboratory. Existing clinical studies have not standardized the inclusion criteria for liquid biopsy candidates, and whether or not to participate in the studies is largely dependent on clinical practice and patients’ wishes. How to select the most suitable candidates for liquid biopsy needs to be explored in robust studies. Most of the clinical trials in existence, on the other hand, have the shortcomings of small patient enrollment, short follow-up time, and single-center origin. Conditions such as sample provenance, enrichment techniques, sampling duration, etc., used in the different studies lacked uniformity. Therefore, relevant comparisons cannot be made. Large, prospective, independent, multicenter cohort studies are needed to validate the current results.

In summary, although the development of liquid biopsy lagged behind other types of malignant tumors. Liquid biopsy has shown quite an advantage in HCC screening, diagnosis, treatment and prognostic monitoring as well, and it has room for development. We can expect that liquid biopsy will emerge as an essential instrument in HCC patient management one day in the future.

## Figures and Tables

**Table 1:** Existing detection technologies for CTCs and the pros and cons of each.

Methodologies	Characterization	Drawbacks	Merits	Reference
physical isolation assay				
Deformability; density; cell size; cell surface electric load	Based on the physical property differences between CTCs and blood cells	Low capture rates Cannot distinguish the source of CTCs	High processing efficiency. Simple and easy to implement	[33,34,35,36]
immunoaffinity isolation assay ^1^				
Positive selection				
Cell Search system ^2^	Magnetic beads coupled with Ep CAM ^3^ bound to ferrofluid-based system	False-negative possibility- CTCs undergoing EMT probably do not express Ep CAM. low purity of captured cells	CTCs can be visualized and quantified under fluorescence microscopy. CTCs isolated can be analyzed for mRNA expression and DNA mutations High sensitivity, detecting approximately 5 CTCs per 7.5 mL whole blood	[42,43,44]
Adna Test	MUC1 and Ep CAM antibody-labeled immunomagnetic beads combined with RT-PCR for detection of tumor-associated transcripts	False-positive possibility—activated leukocytes can express MUC1 Harsh blood sample handling and storage conditions	Promising to detect tumor-specific transcripts not typically detected in nucleated blood cells Highly specific and sensitive (detecting approximately 22 CTCs per 5 mL whole blood)	[45,46]
GILUPI CellCollector™	Invasive enrichment modality—Ep CAM-coated wire placed into patient’s vein for CTCs capture	Invasive Long intravascular presence	Single processing of large volumes of blood. High isolation purity Genetic analysis can be continued	[47]
Negative selection				
Can Patrol™	Negative Enrichment ^4^ combined with filtration based on cell size	Low purity—low levels of leukocyte interference	CTCs without classical epithelial antigen expression were captured	[48]
Rosette Sep™	Recognition and removal WBCs and RBCs from blood samples by antibody complexes (CD45, CD66b and glycoproteins) in combination with flow cytometry	Slow processing speeds Excessive shear forces may affect cell viability and cell ligand attachment.	Higher capture specificity and selectivity The captured CTCs can proceed to phenotyping and molecular analysis	[49,50]
Cyttel method	Negative enrichment modality ^5^ combined with immunofluorescence and fluorescence in situ hybridization.	False positive results Lack of harmonized protocols for clinical application	High recovery rate Enriched cells with good activity High sensitivity and specificity	[51,52]
Combined positive and negative selection				
CTC-iChip	Microfluidic platform using lateral displacement, inertial focusing and magnetophoresis coupled with tumor antigen-dependent and/or non-dependent enrichment of CTCs	Low purity Complexity of production	High separation efficiency and yield Cytopathological and molecular characterization of both epithelial and non-epithelial cancers	[53,54,55]
MACS system	Immune-magnetic CTC enrichment using cell surface marker antibodies or intracellular anti-pan CK antibodies	Identifies Ep CAM-negative cells but not CK-negative ones	High efficiency Can be combined with leukocyte depletion	[56,57]

^1^ Immunoaffinity isolation assay has two main selection methods. Positive selection utilizes tumor-specific markers to select CTCs from the sample. Negative selection achieves CTCs enrichment by removing other cellular components in the sample, and it is not affected by tumor cell surface marker expression variations [58]. ^2^ The only FDA-approved assay to screen CTCs in metastatic breast, colon and prostate carcinoma [45,59,60,61]. ^3^ Epithelial cell adhesion molecule (Ep CAM) has been identified abnormal in a spectrum of malignancies, including HCC [62]. ^4^ The Can Patrol protocol removes erythrocytes by erythrocyte lysis and eliminates CD45+ leukocytes using magnetic bead separation for negative enrichment. ^5^ The Cyttel method utilizes hypotonic hemolysis to clear erythrocytes and anti-CD45 antibody co-coupled magnetic beads to clear leukocytes for negative enrichment.

**Table 2:** Expression of anomalous miRNAs in HCC tissues and the roles of each.

miRNAs	Expression Level	Function	Roles	Reference
miR-182	Upward	Promote HCC proliferation and metastasis Increase HCC resistance to cisplatin	Prediction of recurrence	[108,109,110]
miR-21	Upward	Promote hepatocyte steatosis, inflammation and fibrosis Increase HCC cells proliferation and invasion, inhibit apoptosis	Potential therapeutic targets	[111]
miR-454	Upward	Participate in HCC genesis and tumor stem cell self-renewal Participate in HCC resistant to sorafenib	Prognostic prediction and therapeutic targets related to HCC resistance	[112]
miR-873	Upward	involve in hepatocyte metabolism and HCC occurrence	Prognostic prediction	[113]
miR-18a	Upward	Enhance HCC invasion, migration, and proliferation	Prognostic prediction	[114]
miR-130b-3p	Upward	Promotes HCC angiogenesis	Potential therapeutic target and prognostic markers	[115]
miR-375	Upward	Inhibit HCC angiogenesis Participate in HCC resistant to sorafenib	Potential therapeutic targets related to HCC resistance	[116]
miR-25	Upward	Induce HCC resistance to sorafenib Regulate HCC apoptosis	Potential therapeutic targets related to HCC resistance	[117]
miR-1246	Upward	Involved in HCC development	Potential early detection and prognostic prediction	[118]
miR-296-5p	Downward	Inhibit HCC invasion, migration, and proliferation	Prognostic prediction	[119,120]
miR-206	Downward	Regulate hepatocyte viability, migration and apoptosis Inhibit HCC development	Multifunctional anti-tumor effects	[121]
miR-497	Downward	Involved in HCC development	Potential early detection and prognostic prediction	[118]
miR-106-5p miR-372-5p	Downward	Inhibit HCC proliferation and invasion and promote HCC apoptosis	Potential therapeutic targets related to HCC resistance and prognostic markers	[122]
miR-424-5p	Downward	Inhibit HCC cell proliferation and invasion	Potential therapeutic target and prognostic markers	[123]
miR-3064-5p	Downward	Inhibit HCC angiogenesis	Potential therapeutic target and prognostic markers	[124]
miR-203	Downward	Enhance HCC radiosensitivity	Prognostic prediction and Potential therapeutic target	[125]
miR-146a	Downward	Inhibit HCC proliferation and induce HCC apoptosis Enhance HCC radiosensitivity	Potential therapeutic target	[126]
miR-621	Downward	Enhance HCC radiosensitivity	Potential therapeutic target	[127]
miR-148b	Downward	Promote HCC metastasis	Prognostic prediction and Potential therapeutic target	[128]
miR-144 miR-451a	Downward	Enhance anti-tumor immunity	Prognostic prediction and Potential therapeutic target	[129]

**Table 3:** Materials with HCC-related aberrant expression in exosomes and the roles of each.

Materials	Biomarker	Source	Expression Levels	Function	Reference
Protein					
	LOXL4	HCC cells	Upward	Involved in HCC metastasis	[149]
	MET	HCC cells	Upward	Involved in tumor proliferation, angiogenesis metastasis	[150]
	Triosephosphate isomerase 1 (TPI1)	HCC cells	Downward	Inhibit the development of HCC	[151]
	CAV1	HCC cells	Upward	Involved in HCC formation and metastasis	[150,152,153]
	Pyruvate kinase M2 isoform (PKM2)	HCC cells	Upward	Reshape the Tumor Microenvironment Promote HCC development	[154]
	Alpha-enolase (ENO1)	HCC cells	Upward	Promote HCC growth, metastasis	[155]
	p120-catenin	HCC cells	Downward	Inhibit HCC proliferation and metastasis	[156]
	14-3-3 protein zeta	HCC cells	Upward	Promotes HCC invasion and metastasis Involved in T-lymphocyte depletion	[157]
	Complement Factor H (CFH)	HCC cells	Upward	Promote HCC growth, migration, and invasiveness	[158]
Non-coding RNAs					
	circRNA-PTGR1	HCC cells	Upward	Enhances migration and invasion of low-transfer-potential HCC cells	[159]
	circRNA-100338	HCC cells	Upward	Enhance HCC metastasis promote HCC angiogenesis Prognostic prediction	[160]
	lncRNA FAL1	HCC cells	Upward	Enhance HCC proliferation and migration capacity.	[161]
	miRNA-15b	macrophages	Upward	Promote HCC proliferation, migration and invasion	[162]
	miR-103	HCC cells	Upward	Increase vascular permeability promote HCC metastasis	[163]
	miR-584-5p	HCC cells	Upward	Promote HCC angiogenesis	[164]
Non-coding RNAs					
	miR-210	HCC cells	Upward	Promote HCC angiogenesis	[165]
	miR-21 miR-10b	HCC cells	Upward	Promote HCC proliferation, migration and invasion Prognostic prediction	[166,167]
	miR-1247-3p	HCC cells	Upward	Related to HCC lung metastasis	[168]
	miR-92b	HCC cells	Upward	Enhance HCC migration Down-regulated natural killer (NK) cell-mediated cytotoxicity Prognostic prediction	[169]
	miR-23a-3p	HCC cells	Upward	Upregulate PD-L1 expression in macrophages to help HCC evade anti-tumor immunity.	[170]

**Table 4:** Application of liquid biopsy in the management of HCC.

	CTCs	cfDNA	ctDNA	Circulating microRNA	Exosomes	TEPs
Screening	Need for research	Need for research	√	√	√	√
Diagnosis	Need for research	Need for research	√	√	√	Need for research
Treatment	√	√	√	√	√	Need for research
Prognostic monitoring	√	√	√	√	√	Need for research

## Data Availability

Not applicable.

## Abbreviations

AR	anatomical resection
BCLC	barcelona clinic liver cancer
CTC	circulating tumor cell
cfDNA	circulating cell-free DNA
CSC	tumor stem cell
ctDNA	circulating tumor DNA
dd PCR	droplet digital PCR
DN	dysplastic nodule
E-CTC	epithelial circulating tumor cell
Ep CAM	epithelial cell adhesion molecule
E/M-CTC	intermediate subtypes undergoing transformation circulating tumor cell
EMT	epithelial mesenchymal transition
EV	extracellular vesicle
FDA	US Food and Drug Administration
HBV	hepatitis B virus
HCC	hepatocellular carcinoma
HCV	hepatitis C virus
I FISH	immunostaining-fluorescence in situ hybridization
LDLT	living donor liver transplantation
LT	liver transplantation
M-CTC	mesenchymal circulating tumor cell
mRNA	messenger RNA
MVI	microvascular infiltration
NAR	non-anatomical resection
NGS	next-generation sequencing
NSCLC	non-small cell lung cancer
qPCR	quantitative PCR
RFA	radiofrequency ablation
TA	thermal ablation
TACE	transcatheter arterial chemoembolization
TEP	tumor-educated platelets
WGS	Whole Genome Sequencing
